# Silicon‐Based Agent Attenuates Age‐Related Spermatogenesis Disorder

**DOI:** 10.1111/iju.70189

**Published:** 2025-07-29

**Authors:** Sohei Kuribayashi, Shinichiro Fukuhara, Hiroaki Kitakaze, Go Tsujimura, Takahiro Imanaka, Norichika Ueda, Kentaro Takezawa, Hikaru Kobayashi, Ryoichi Imamura, Norio Nonomura

**Affiliations:** ^1^ Department of Urology Osaka University Graduate School of Medicine Suita Japan; ^2^ Research Institute for Microbial Diseases Osaka University Suita Japan; ^3^ Department of Urology Glickman Urological & Kidney Institute, Cleveland Clinic Foundation Cleveland Ohio USA; ^4^ Department of Urology JCHO Osaka Hospital Osaka Japan; ^5^ SANKEN (Institute of Scientific and Industrial Research), Osaka University Osaka Japan; ^6^ Department of Urology Nagasaki University Graduate School of Biomedical Science Nagasaki Japan

**Keywords:** age, antioxidant, hydrogen, male infertility, silicon, spermatogenesis

## Abstract

**Objectives:**

The progressive decline in birth rates due to delayed marriage has become a significant issue in developed countries, with male spermatogenetic capabilities gradually declining with age. This study evaluated the effectiveness of a silicon‐based agent (Si‐based) that efficiently generates hydrogen in the body to improve spermatogenic function in aged mice.

**Methods:**

Eighty‐five‐week‐old (aged) C57BL/6J mice were administered either a normal diet or a diet supplemented with a Si‐based agent. Sperm analysis, histopathology, immunohistochemistry, immunofluorescence, and immunoblotting were performed to assess spermatogenic function, markers of oxidative stress, and activation of the Kelch‐like ECH‐associated protein 1 (KEAP1)/nuclear factor erythroid 2‐related factor 2 (NRF2) pathway.

**Results:**

The Si‐based agent significantly increased sperm count and velocity (curvilinear and straight‐line velocity), reduced the proportion of abnormal seminiferous tubules, and improved testicular histology in aged mice (*p* < 0.05). Furthermore, the Si‐based agent decreased the expression of oxidative stress markers and modulated the KEAP1/NRF2 pathway by reducing the expression of KEAP1 and increasing NRF2 nuclear translocation, leading to the upregulation of the antioxidant genes—heme oxygenase (HMOX)‐1 and NAD(P)H quinone oxidoreductase (NQO‐1).

**Conclusion:**

These findings suggest that hydrogen delivered through a Si‐based agent could be a promising therapeutic approach for age‐related male infertility. This warrants further studies to explore its potential in clinical settings.

## 1 Introduction

The declining birth rate in developed countries has become a major concern, largely attributed to delayed marriage [1]. In men, spermatogenesis gradually deteriorates with age [2], a process accompanied by increased oxidative stress and the accumulation of reactive oxygen species (ROS), which contribute to the onset of various diseases [3]. Although various antioxidants have been explored as treatments for age‐related male infertility [4, 5], there remains a lack of strong evidence supporting their clinical efficacy.

Hydrogen is a medical gas recognized for its anti‐inflammatory, antioxidant, and antiapoptotic properties, along with a well‐established safety profile [6]. It has been shown to protect cells and tissues from oxidative stress and inflammation‐induced damage. Various methods of hydrogen administration, including inhalation, consumption of drinking hydrogen‐rich water, and intraperitoneal injection of hydrogen‐rich saline, have demonstrated efficacy in mitigating ischemia–reperfusion injury [7], sepsis [8], diabetes [9], radiation injury [10], and other diseases [11]. However, the limited absorption efficiency of hydrogen when administered in gaseous or dissolved forms presents a significant challenge.

To overcome this issue, we successfully developed a silicon‐based agent (Si‐based agent) that efficiently generated hydrogen in the body. The agent reacts with water in the pH range of 7.0–8.6, leading to substantial hydrogen release [12]. The rate of hydrogen generation is highly dependent on the crystallite size of the particles and the pH of the surrounding environment. Notably, nano‐Si particles with a median diameter of 9.6 nm produced up to 55 mL of hydrogen within 1 h in water with a pH of 8.0, which is equivalent to the hydrogen content in approximately 3 L of saturated hydrogen‐rich water. The therapeutic potential of Si‐based agents has been demonstrated in models of kidney transplant rejection [13] and varicocele‐related infertility [14].

Given these findings, this study aimed to evaluate the efficacy of a Si‐based agent in alleviating age‐related male infertility and to investigate the underlying mechanisms by which hydrogen administration enhances spermatogenic function.

## 2 Materials and Methods

### 2.1 Animals

Aged C57BL/6J mice (85 weeks old) were housed under standard laboratory conditions (22°C, 12/12‐h light/dark cycles). They were purchased from Jackson Laboratories Japan.

### 2.2 Ethics Statement

All animal procedures were approved by the Institutional Animal Care and Use Committee of Osaka University (IACUC approval no.: J008270‐003).

### 2.3 Supplementation of the Si‐Based Agent in Food

AIN93M (Oriental Yeast Co. Ltd., Tokyo, Japan) was used as a standard diet. To evaluate the effect of the Si‐based agent, a modified diet containing 1.0 wt% of the agent was administered starting at 85 weeks of age and continued until killing at 90 weeks of age, for a total of 5 weeks of treatment. The duration was selected based on the spermatogenic cycle in mice, which typically takes approximately 35 days.

### 2.4 Sperm Analysis

Spermatozoa were collected from the cauda epididymis and incubated in Toyoda, Yokoyama, and Hoshi (TYH) medium. After incubation for 30 min in TYH medium, spermatozoa were obtained from the top of the drops and analyzed using the CEROS II (v.1.4; Hamilton Thorne Biosciences) sperm analysis system.

### 2.5 Histopathology

Testes were fixed in 10% formalin, embedded in paraffin, and sectioned into 4‐μm‐thick slices. These sections were stained with hematoxylin and eosin and analyzed using a BZ‐X700 microscope (Keyence Co., Osaka, Japan). Seminiferous tubules exhibiting lumen disorganization, vacuolization, or germ cell depletion were classified as abnormal. The abnormality rate was assessed at 40× magnification and defined as:

$$\text{Abnormality rate}=\frac{\text{Number of abnormal seminiferous tubules}\;\mathrm{per}\;\text{field of view}}{\text{Total number of seminiferous tubules}}$$

Johnsen's score analysis. For each animal, 100 randomly selected seminiferous tubules were examined on hematoxylin–eosin‐stained sections. Tubules were scored on the 1–10 Johnsen scale, and the mean score per mouse was used for statistical analysis.

### 2.6 Immunohistochemistry

For immunohistochemical analysis, tissue sections that had been fixed in formalin and embedded in paraffin were deparaffinized. Antigen retrieval was conducted using a pressure cooker in citrate buffer (pH 6), and endogenous peroxidase activity was inhibited using Dako REAL Peroxidase‐Blocking Solution (Agilent, CA, USA). The sections were then incubated overnight at 4°C with primary antibodies diluted in Dako Antibody Diluent (Agilent). Immunostaining was performed using the Dako EnVision+ System HRP‐Labeled Polymer Anti‐rabbit (Agilent) and Dako Liquid DAB+ Substrate Chromogen System (Agilent) following the manufacturers' protocols. After staining, the sections were counterstained with hematoxylin and examined using a BZ‐X700 microscope.

The primary antibodies used included rabbit anti‐4‐hydroxy‐2‐nonenal (anti‐4HNE; 1:300; # bs‐6313R, Bioss Antibodies Inc., MA, USA) and rabbit anti‐Ki67 (1:300; # ab16667; Abcam, MA, USA).

Ki67 immunostaining was quantified on paraffin sections with a BZ‐X Analyzer (Keyence Co.) at ×20 magnification. For each mouse, one centrally located field from a mid‐testicular section—containing several thousand nuclei—was analyzed. Ki67‐positive nuclei were automatically identified with a fixed threshold, and the Ki67‐positive rate was calculated as (Ki67‐positive nuclei/total nuclei) × 100%.

4‐HNE staining was evaluated per seminiferous tubule on a semi‐quantitative 0–2 scale: 0 (no staining), 1 (mild staining, characterized by faint staining of the cytoplasm and nuclei in seminiferous tubules), and 2 (strong staining, with intense cytoplasmic and nuclear staining in seminiferous tubules). Twenty tubules per mouse were scored; the mean score per mouse was used for statistical analysis.

### 2.7 Immunofluorescence

Immunofluorescence analysis was conducted on 4‐μm‐thick serial sections of formalin‐fixed, paraffin‐embedded tissues. The sections were first deparaffinized and subjected to antigen retrieval using a pressure cooker in citrate buffer (pH 6). Following this, primary antibodies were applied and incubated overnight at 4°C. The next day, the sections were incubated with secondary antibodies for 60 min at room temperature (20°C–25°C). After washing with Tris‐buffered saline containing Tween 20 (Merck, Darmstadt, Germany), the slides were counterstained using Vectashield Hardset Mounting Medium (Vector Laboratories, CA, USA). The stained sections were then analyzed under a BZ‐X700 microscope. For immunolabeling, rabbit anti‐nuclear factor erythroid 2‐related factor 2 (NRF2; 1:300; ab31163; Abcam) was used as the primary antibody, while Alexa Fluor 488‐conjugated anti‐rabbit antibody (1:300; #A11034; Thermo Fisher Scientific, MA, USA) was used as the secondary antibody.

### 2.8 Immunoblotting

Proteins were extracted from whole testes by homogenizing in RIPA buffer (Nacalai Tesque, Kyoto, Japan) supplemented with protease inhibitors (Nacalai Tesque), followed by the collection of whole lysates. Protein concentrations were determined using the Lowry's method [15]. The extracted proteins were stored at −80°C until further analysis. For immunoblotting, 20 μg of protein was subjected to 10% sodium dodecyl sulfate‐polyacrylamide gel electrophoresis (SDS‐PAGE) and transferred onto membranes. The membranes were blocked with Bullet Blocking One for Western Blotting (Nacalai Tesque) and incubated overnight at 4°C with primary antibodies diluted in Tris‐buffered saline. Detection was performed using horseradish peroxidase (HRP)‐conjugated anti‐rabbit immunoglobulin (IgG) antibody (Cell Signaling Technology, MA, USA) and Chemi‐Lumi One (Nacalai Tesque). Blot images were captured using a ChemiDoc XRS Plus imaging system (Bio‐Rad, CA, USA), and band quantification was conducted with ImageJ software (National Institutes of Health, MD, USA). The primary antibodies used were as follows: rabbit anti‐Kelch‐like ECH‐associated protein 1 (KEAP1; 1:1000; #8047S; Cell Signaling Technology), rabbit anti‐heme oxygenase 1 (HMOX1; 1:1000; #86806S; Cell Signaling Technology), rabbit anti‐NAD(P)H quinone dehydrogenase 1 (NQO‐1; 1:1000; # ab80588; Abcam), rabbit anti‐nuclear factor erythroid 2‐related factor 2 (NRF2; 1:1000; #12721S; Cell Signaling Technology), and rabbit anti‐actin beta (ACTB; 1:1000; #4967S; Cell Signaling Technology). The secondary antibody used was HRP‐conjugated anti‐rabbit IgG antibody (1:5000; #7074; Cell Signaling Technology).

### 2.9 Statistical Analysis

All data were analyzed using JMP 17 (SAS Institute Inc., Cary, NC, USA). All data are presented as the mean ± standard deviation, and *p* < 0.05 were considered statistically significant. A two‐tailed Student's *t*‐test for experiments with two groups was used for analysis, as appropriate, where * indicated *p* < 0.05.

## 3 Results

### 3.1 The Si‐Based Agent Enhanced Sperm Production in Aged Mice

**FIGURE 1:**
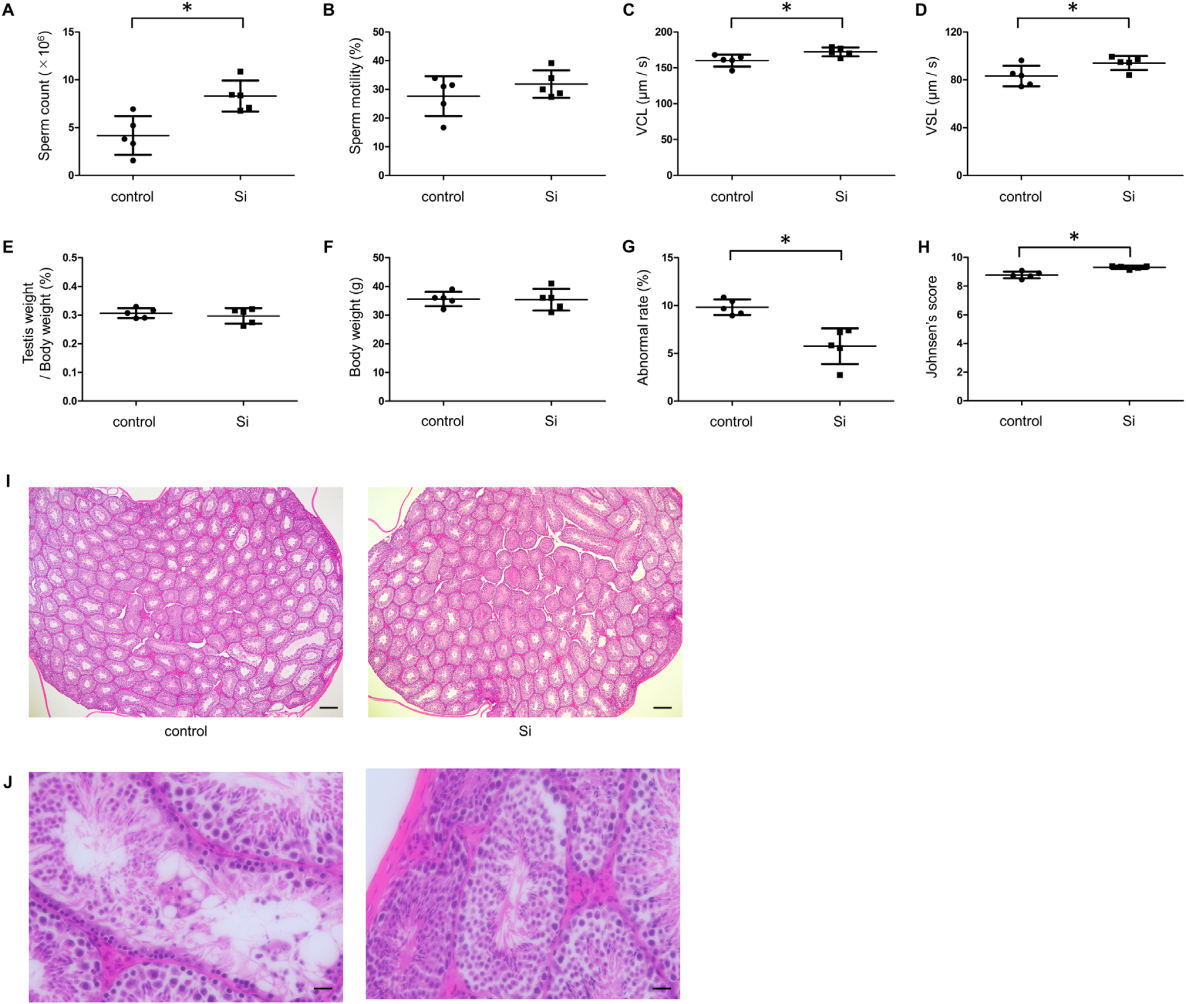
Effect of Si‐based agent on spermatogenesis. (A) Sperm count, (B) sperm motility, (C) VCL, (D) VSL, (E) testis weight/body weight, (F) body weight, (G) abnormality rate, and (H) Johnsen's score in the control and Si‐based agent (Si) groups (*n* = 5 per group). (I) Hematoxylin and eosin staining of the testes in the two groups. (J) Representative higher magnification images of seminiferous tubules in the control group. Left: Vacuolization; right: Lumen disorganization. Data are expressed as the mean ± SD. All statistical analyses were performed using a two‐tailed Student's *t*‐test. Scale bars: 200 μm (I) and 20 μm (J). SD, standard deviation; Si, silicon; VCL, curvilinear velocity; VSL, straight line velocity.

Administration of the Si‐based agent led to a significant increase in sperm count compared to the control group (control vs. Si: 4.17 × 10^6^ vs. 8.30 × 10^6^, *p* = 0.0074, Figure 1A). This indicated a notable improvement in spermatogenesis in aged mice. Additionally, while the increase in sperm motility did not reach statistical significance, there was a trend toward increased sperm motility (control vs. Si: 27.6% vs. 31.8%, *p* = 0.2948, Figure 1B), suggesting a potential benefit for sperm functionality.

Further analysis revealed that the Si‐based agent significantly enhanced curvilinear velocity (control vs. Si: 160 vs. 172 μm/s, *p* = 0.0318, Figure 1C) and straight‐line velocity (control vs. Si: 83.1 vs. 94.0 μm/s, *p* = 0.0478, Figure 1D), both of which are key parameters for sperm motility and fertilization potential. In contrast, testicular weight remained unchanged between the two groups (control vs. Si: 0.30% vs. 0.30%, *p* = 0.5233, Figure 1E). Furthermore, there was no significant difference in body weight between the groups throughout the treatment period (Figure 1F). Aging is commonly associated with an increase in seminiferous tubule abnormality. Si‐based agent treatment significantly reduced the proportion of abnormal seminiferous tubules (control vs. Si: 8.33% vs. 4.26%, *p* = 0.0021, Figure 1G,I,J) and increased the mean Johnsen's score (control vs. Si: 8.77 vs. 9.31, *p* = 0.0017, Figure 1H), indicating a protective effect on testicular histology.

### 3.2 The Si‐Based Agent Alleviates Oxidative Stress in Seminiferous Tubules via KEAP1/NRF2 Pathway Activation

**FIGURE 2:**
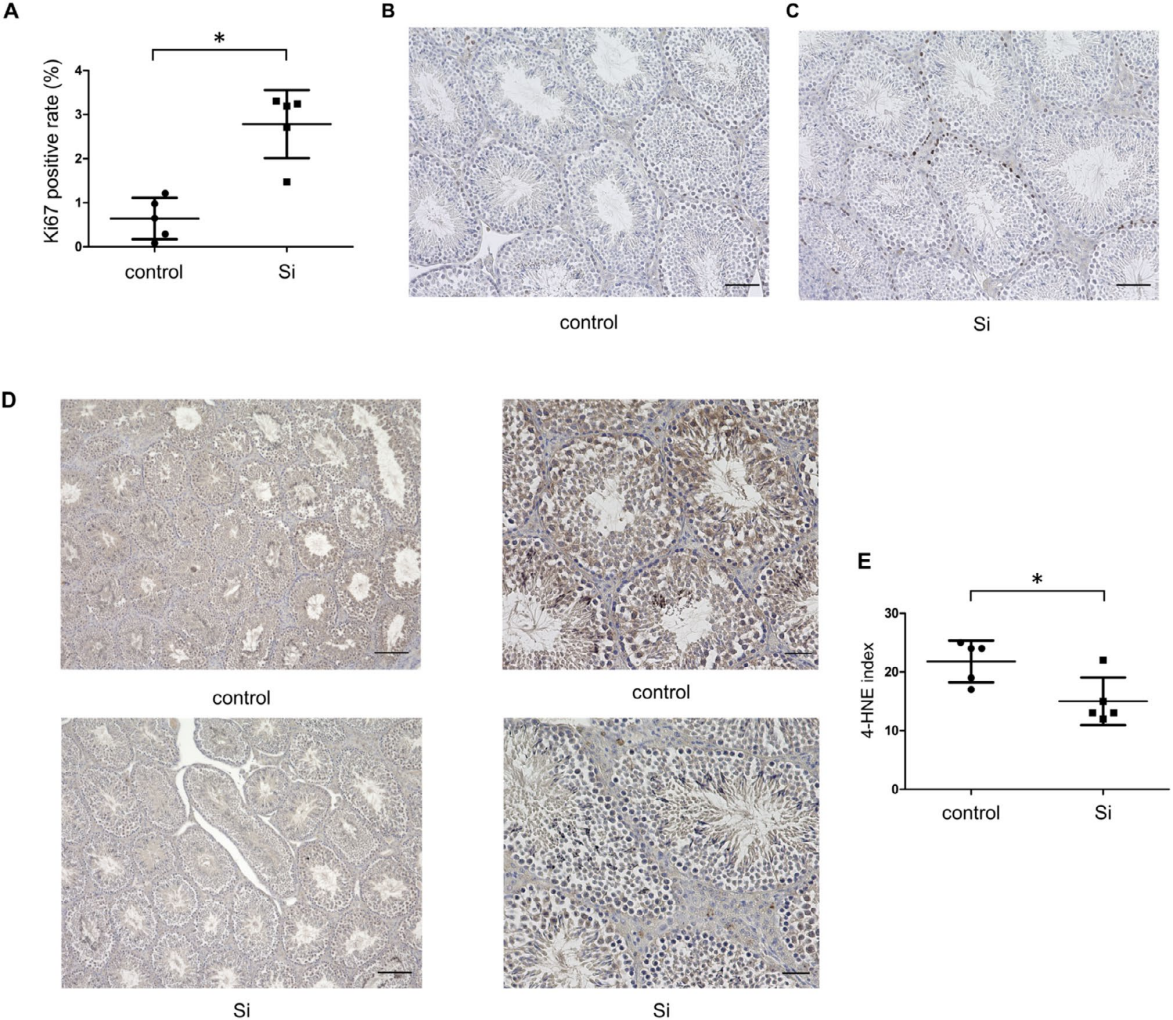
Effect of Si‐based agent on cell proliferation and oxidative stress. (A) Ki‐67 positivity rates in individual seminiferous tubules of mice in the two groups (*n* = 5 per group). (B) Ki‐67 immunohistochemistry of the control testes. (C) Ki‐67 immunohistochemistry of the Si‐treated testes. (D) 4‐HNE immunohistochemistry of the testes in the two groups. (E) 4‐HNE index of the mice in the two groups (*n* = 5 per group). Data are expressed as the mean ± SD. All statistical analyses were performed using a two‐tailed Student's *t*‐test. Scale bars: 100 μm (D, left), 50 μm (B, C, and D right). 4HNE, 4‐hydroxy‐2‐nonenal; SD, standard deviation.

The Si‐based agent significantly upregulated Ki67 expression (control vs. Si: 0.64% vs. 2.78%, *p* = 0.0007, Figure 2A–C), a marker of cell proliferation, suggesting enhanced cellular activity in the testes. Moreover, the oxidative stress marker, as indicated by 4‐HNE expression, was significantly reduced following treatment (control vs. Si: 21.8 vs. 15.0, *p* = 0.0007, Figure 2D,E), highlighting a reduction in oxidative damage within the seminiferous tubules.

**FIGURE 3:**
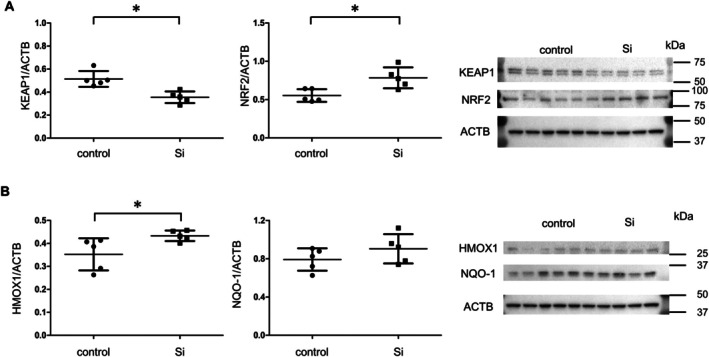
Effect of Si‐based agent on oxidative stress‐related genes. (A) Representative immunoblotting (right) and quantitative analysis (left) of KEAP1, NRF2, and ACTB in the testes of mice in the two groups (*n* = 5 per group). (B) Representative immunoblotting (right) and quantitative analysis of HMOX1 (left), NQO‐1 (middle), and ACTB in the two groups (*n* = 5 per group). Data are expressed as the mean ± SD. All statistical analyses were performed using a two‐tailed Student's *t*‐test. ACTB, actin beta; HMOX1, heme oxygenase 1; KEAP1, Kelch‐like ECH‐associated protein 1; NQO‐1, NAD(P)H quinone dehydrogenase 1; NRF2, nuclear factor erythroid 2‐related factor 2; Si, silicon.

Regarding the KEAP1/NRF2 oxidative stress response pathway, the Si‐based agent led to a significant decrease in KEAP1 expression (control vs. Si: 0.514 vs. 0.355, *p* = 0.0030, Figure 3A), a molecule that is typically upregulated with aging. Additionally, NRF2 protein expression was significantly elevated in Si‐treated testes (control vs. Si: 0.553 vs. 0.785, *p* = 0.0116, Figure 3A), consistent with activation of downstream antioxidant signaling. This activation resulted in a significant upregulation of HMOX‐1, an NRF2 target gene involved in oxidative stress defense (control vs. Si: 0.352 vs. 0.433, *p* = 0.04, Figure 3B). While the expression of NQO‐1, another NRF2‐regulated antioxidant gene, showed an increasing trend (control vs. Si: 0.791 vs. 0.903, *p* = 0.2291 Figure 3B), it did not reach statistical significance. These findings indicate that the Si‐based agent mitigates oxidative stress by activating the NRF2 pathway, thereby supporting a healthier spermatogenic environment in aged mice.

## 4 Discussion

In this study, we demonstrated that the administration of a Si‐based agent considerably attenuated spermatogenesis in aged mice. Treatment with the Si‐based agent led to increased sperm count and motility parameters, a reduction in the proportion of abnormal seminiferous tubules, and a decrease in oxidative stress markers, such as 4‐HNE. These effects were linked to the activation of the KEAP1/NRF2 pathway, suggesting a protective role of the Si‐based agent against age‐related testicular dysfunction.

Eighty‐five‐week‐old C57BL/6J males were chosen to model late‐stage testicular aging, a stage at which spermatogenesis is markedly impaired. Histological surveys show that by 18–24 months, many seminiferous tubules are depleted of germ cells and epididymal sperm counts fall by > 50% [16]. Using such an advanced age provides a stringent context to test whether antioxidant therapy retains efficacy when the spermatogenic niche is already compromised. Although this age exceeds the typical reproductive span in mice, it approximates the human equivalent of 60 years and has been widely adopted in aging studies of the reproductive, nervous, and cardiovascular systems. Future work will evaluate earlier aging stages (12–15 months) to determine how soon Si‐based treatment should begin for maximal benefit.

Age‐related dysfunction of spermatogenesis is influenced by multiple interconnected mechanisms [17]. Among these, oxidative stress plays a major role, as excessive reactive oxygen species (ROS) in the testes contribute to DNA damage and impaired sperm function [18]. While physiological levels of ROS are essential for sperm capacitation and other key processes [19], excessive ROS leads to sperm DNA fragmentation and mutations, ultimately compromising fertility and genetic integrity [18]. Additionally, dysfunction in spermatogonial stem cells reduces spermatogenic capacity over time [20], while age‐related changes in Sertoli and Leydig cell function, along with testicular atrophy, further impair sperm production and maturation [16].

Various antioxidants, including coenzyme Q10 [21], vitamins C [22] and E [23], and zinc [24] have been investigated for their ability to reduce oxidative stress and support spermatogenesis. However, clinical evidence supporting their effectiveness remains limited. Hydrogen possesses strong antioxidant properties [6], yet a reliable method for its sustained generation within the body has been lacking. To address this, we developed a Si‐based agent capable of safely producing hydrogen in vivo [12], with prior studies demonstrating its efficacy in ischemia–reperfusion injury [13] and varicocele models [14]. Although the Si‐based agent itself does not enter the circulation, the hydrogen produced in the gut is rapidly absorbed, circulates systemically, and—because of its small, nonpolar nature—can permeate the blood–testis barrier (BTB). Consistent with our previous finding that Si‐based treatment elevates systemic H_2_ levels [13], Liu et al. [25] have shown that exogenously delivered hydrogen—via hydrogen‐rich water, hydrogen‐rich saline, or inhaled hydrogen gas—reaches micromolar concentrations in rodent testes within minutes, as measured by needle‐type electrodes or gas chromatography. In line with this report, the present study showed reduced oxidative stress and upregulation of NRF2‐dependent antioxidant genes following Si‐based treatment, even though direct quantification of intratesticular H_2_ was not feasible with our current detection limit. Future work will employ more sensitive microsensors or mass‐spectrometric methods to confirm H_2_ concentrations in aged testis.

The KEAP1/NRF2 pathway is a key regulator of cellular defense against oxidative stress [26]. Under normal conditions, NRF2 remains sequestered in the cytoplasm through its interaction with KEAP1. However, oxidative stress triggers modification of KEAP1's cysteine residues, leading to the release of NRF2, which translocates to the nucleus and activates antioxidant genes such as HMOX‐1 and NQO‐1, thereby mitigating oxidative stress. Aging has been associated with increased KEAP1 expression in the testes, impairing NRF2‐dependent antioxidant responses [27]. Our findings indicate that treatment with the Si‐based agent reduced KEAP1 protein levels and increased total NRF2 expression, which was accompanied by upregulation of the NRF2 target gene HMOX‐1. Although nuclear enrichment of NRF2 could not be unequivocally demonstrated, these data indicate activation of the KEAP1/NRF2 axis and suggest that the Si‐based agent enhances intrinsic antioxidant defenses, thereby supporting spermatogenesis in aged testes.

Despite these promising findings, our study has certain limitations. First, individual food intake was not recorded, which precluded precise estimation of the actual dose of the Si‐based agent administered to each mouse. Nonetheless, body weight remained stable throughout the treatment period (Figure 1F), suggesting that the diet was well tolerated and did not affect overall health status. Second, the use of an animal model, while informative, may not fully represent human physiology. Further studies in human subjects are necessary to validate these results and assess the long‐term safety and efficacy of Si‐based agents. Third, we were unable to confirm NRF2 nuclear translocation owing to insufficient nuclear protein yields and limited immunofluorescence resolution. Future studies employing optimized fractionation will be required to validate NRF2 nuclear import in response to the Si‐based agent.

Further research should prioritize clinical trials to evaluate the potential of Si‐based agents as a therapeutic strategy for age‐related male infertility in humans. Additionally, investigating the broader application of this agent in other age‐associated conditions, as well as its interactions with oxidative stress and inflammatory pathways, may offer further insights into its therapeutic potential.

## Author Contributions

**Sohei Kuribayashi:** conceptualization, methodology, software, data curation, investigation, writing – original draft, writing – review and editing, formal analysis, validation. **Shinichiro Fukuhara:** conceptualization, formal analysis, project administration, writing – review and editing. **Hiroaki Kitakaze:** formal analysis, data curation, visualization. **Go Tsujimura:** data curation. **Takahiro Imanaka:** data curation. **Norichika Ueda:** methodology. **Kentaro Takezawa:** methodology. **Hikaru Kobayashi:** project administration. **Ryoichi Imamura:** project administration, resources. **Norio Nonomura:** writing – review and editing, supervision.

## Disclosure

Approval of the Research Protocol by an Institutional Review Board: The authors have nothing to report.

Registry and the Registration No. of the Study/Trial: The authors have nothing to report.

Animal Studies: All animal procedures were approved by the Institutional Animal Care and Use Committee of Osaka University (IACUC approval no.: J008270‐003).

## Consent

The authors have nothing to report.

## Conflicts of Interest

Norio Nonomura and Kentaro Takezawa are the Editorial Board members of the *International Journal of Urology* and a coauthors of this article. To minimize bias, they were excluded from all editorial decision‐making related to the acceptance of this article for publication.

## Data Availability

The authors have nothing to report.
